# A grooved conduit combined with decellularized tissues for peripheral nerve regeneration

**DOI:** 10.1007/s10856-023-06737-z

**Published:** 2023-07-21

**Authors:** Enxing Yu, Zhiwu Chen, Yuye Huang, Yibing Wu, Zonghuan Wang, Fangfang Wang, Miaoben Wu, Kailei Xu, Wei Peng

**Affiliations:** 1 Department of Plastic and reconstructive surgery, The First Affiliated Hospital, Ningbo University School of Medicine, Ningbo, Zhejiang 315010 China; 2 Center for Medical and Engineering Innovation, Central Laboratory, The First Affiliated Hospital, Ningbo University School of Medicine, Ningbo, Zhejiang 315010 China; 3 Central Laboratory, The First Affiliated Hospital, Ningbo University School of Medicine, Ningbo, Zhejiang 315010 China; 4 School of Medicine, Ningbo University, Ningbo, Zhejiang 315211 China; 5 Key Laboratory of Precision Medicine for Atherosclerotic Diseases of Zhejiang Province, Ningbo, Zhejiang 315010 China

**Keywords:** Peripheral nerve injury, Composite nerve conduit, PLGA, Decellularized tissue, Nerve repair, Tissue engineering

## Abstract

Peripheral nerve injury (PNI) is a common and severe clinical disease worldwide, which leads to a poor prognosis because of the complicated treatments and high morbidity. Autologous nerve grafting as the gold standard still cannot meet the needs of clinical nerve transplantation because of its low availability and limited size. The development of artificial nerve conduits was led to a novel direction for PNI treatment, while most of the currently developed artificial nerve conduits was lack biochemical cues to promote nerve regeneration. In this study, we designed a novel composite neural conduit by inserting decellularized the rat sciatic nerve or kidney in a poly (lactic-co-glycolic acid) (PLGA) grooved conduit. The nerve regeneration effect of all samples was analyzed using rat sciatic nerve defect model, where decellularized tissues and grooved PLGA conduit alone were used as controls. The degree of nerve regeneration was evaluated using the motor function, gastrocnemius recovery, and morphological and histological assessments suggested that the combination of a grooved conduit with decellularized tissues significantly promoted nerve regeneration compared with decellularized tissues and PLGA conduit alone. It is worth to note that the grooved conduits containing decellularized nerves have a promotive effect similar to that of autologous nerve grafting, suggesting that it could be an artificial nerve conduit used for clinical practice in the future.

**Graphical Abstract:**

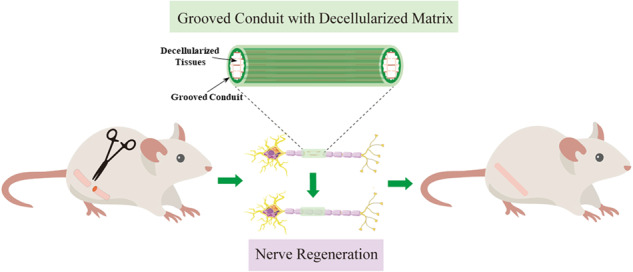

## Introduction

Peripheral nerve injury (PNI) is a common and severe clinical disease, accounting for 2.8% of all trauma cases, and can be caused by tumor removal or surgical procedures [1–3]. PNI commonly leads to a poor prognosis because of the complicated treatments and high morbidity. Most patients with PNI end up with poor rehabilitation of exercise abilities or need long-term care. Currently, the gold standard treatment for PNI is autologous nerve grafting [4] using less important nerves, such as the cutaneous nerve. However, it still cannot meet the needs of clinical nerve transplantation because of its low availability and limited size [5]. Furthermore, autologous nerve transplantation can cause permanent neurological dysfunction at the donor site, which causes more complications [6, 7]. Allogeneic nerve transplantation could be an alternative for PNI repair; however, its widespread clinical application has been prohibited because of immunosuppression [8].

Tissue engineering, such as nerve conduits, has been increasingly considered a potential alternative to nerve autografts for PNI repair [9]. In 1944, Weiss et al. proposed the concept of tissue-engineered neural conduits, which have a complex tubular structure composed of biocompatible materials [10] that connect the two ends of PNI. In 1984, Longo et al. proposed the theory of “nerve regeneration chamber,” believing that reserving an appropriate space between the two ends of PNI has better reparative effects than direct nerve anastomosis, which further promotes the development of nerve conduits because they can guide axons to migrate through the conduits for nerve regeneration while protecting the nerve ends [11]. After decades of development, an ideal nerve conduit should meet the following criteria: (1) it provides sufficient mechanical strength against the compression forces from surrounding tissues and protects the regenerated nerves; (2) it guides regenerated axons to migrate from proximal to distal nerve trunks; (3) it supports the aggregation of neurotrophic factors and promotes nerve regeneration; and (4) it minimizes the formation of scar tissues.

Nerve conduits based on collagen, poly(lactic-co-glycolic acid) (PLGA), and polylactide-ε-caprolactone have achieved US Food and Drug Administration approval for clinical application. PLGA is a commonly used artificial biomaterial that has beneficial features of biocompatibility and biodegradability [12]. Besides, PLGA maintains adequate mechanical support for nerve regeneration [13], which has been investigated in numerous studies [14, 15]. These results suggest that PLGA is dependable as a nerve conduit component. There is a new trend in the application of the natural decellularized extracellular matrix (dECM) as nerve conduit materials [16, 17]. The ECM has the most similar structural, biochemical, and biophysical properties to native tissues, allowing adequate mechanical support for nerve generation [18]. Moreover, it is expected to furnish a suitable microenvironment for the proliferation and maturation of Schwann cells and the regeneration of axons [19]. The dECM retains the ligands for cellular receptor recognition and active growth factors that enhance the cellular adhesion and growth, which makes it a promising material for nerve conduits to support and guide nerve regeneration [20].

**Fig. 1:**
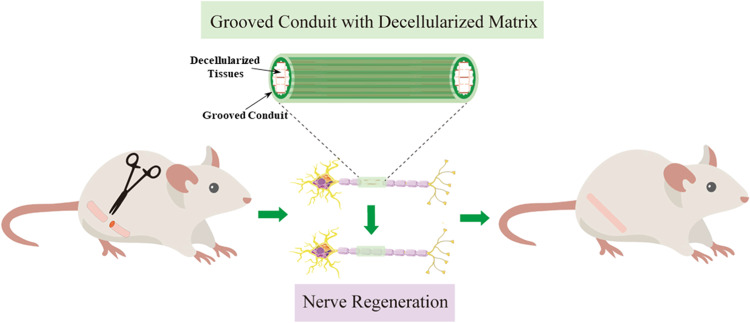
Schematic diagram of the experimental process

In our previous study, PLGA grooved conduits (GCs) fabricated using dry-jet wet electrospinning were used to repair rat PNI [21, 22]. GCs significantly improved the regeneration of peripheral nerves by introducing physical guidance cues compared with smooth conduits (SCs) [23]. Although GCs showed their potential application in nerve regeneration, they still have a significant weakness compared with autografts, which is the lack of biochemical cues. Therefore, we designed a composite nerve conduit by inserting the decellularized rat nerves and kidney in PLGA conduits (Fig. 1). The decellularized kidney was chosen due to the enrichment of erythropoietin (EPO), which is a hormone commonly produced in the kidney that promotes the axonal regeneration in the optic nerve [24] and promotes peripheral nerve regeneration in rats by upregulating the expression of insulin-like growth factor-1 [25]. Our hypothesis is that the inclusion of the decellularized tissues in grooved conduits should significantly improve the outcome of peripheral nerve regeneration.

The physical properties and biocompatibility of PLGA conduits were analyzed before animal experiments. Then, grooved PLGA conduits were filled with decellularized kidney or nerve to compare with decellularized tissues only, grooved PLGA conduit only, and autograft. The rat sciatic nerve regeneration after 16 weeks was evaluated with motor function, gastrocnemius recovery, and morphological and histological assessments, showing that the decellularized nerve could better promote the nerve regeneration compared with decellularized kidney, and the combination of grooved PLGA conduit with decellularized tissues significantly promoted nerve regeneration compared with decellularized tissues and PLGA conduit alone. Although the autograft still has the best promotive effect, grooved PLGA conduit inserted with decellularized nerve demonstrated relatively closer effects, suggesting that it has the potential to be further modified and used as an artificial nerve conduit for clinical practice.

## Materials and methods

### Materials

PLGA was purchased from Medical Instruments Co., Ltd. (Shandong, China). Highly differentiated PC-12 cells were purchased from the Cell Bank of the Chinese Academy of Sciences (Shanghai, China). Fetal bovine serum (FBS), Roswell Park Memorial Institute medium (RPMI-1640), antibiotics (i.e., penicillin, streptomycin, and gentamicin), and amphotericin B were obtained from Gibco (Big Cabin, OK). CCK-8 was purchased from Beyotime (Shanghai, China). The Live/Dead Assay, H&E, Masson’s staining, DNA extraction kits, 40,6-diamidino-2-phenylindole (DAPI), bovine serum albumin (BSA), DAB substrate solution, and phalloidin-fluorescein isothiocyanate were purchased from Solarbio (Beijing, China). Dimethyl sulfoxide (DMSO), sodium dodecyl sulfate (SDS), TritonX-100, heparin sodium solution, and other conventional reagents were purchased from Boyun Biological (Shanghai, China). The TIANamp Genomic DNA Kit was purchased from Tiangen Biochemical Technology Co. (Beijing, China). Furthermore, 9–0 microsutures were purchased from Johnson & Johnson (San Lorenzo, Puerto Rico). Anti-neurofilament 200 antibody (N5389) was purchased from Sigma (St. Louis, MO). Goat anti-mouse antibody (BL001A) was purchased from Biosharp (Anhui, China). Goat serum, anti-S100β (ab52642), anti-neurofilament heavy polypeptide (NF-H, ab19386), and Alexa Fluor 647 (ab150115)/488 (ab150077) were purchased from Abcam (Cambridge, England).

### PLGA conduit fabrication

Nerve conduits were prepared using the dry-jet wet spinning by adapting a previously published method. [21, 26] Briefly, the dope fluid was prepared by mixing PLGA and DMSO solvents with concentration of 17 w/w% for both SC and GC. Deionized (DI) water was used as the spinning bore fluid and pumped into the spinneret. The hollow fiber membranes were prepared with free fall methods and kept in DI water for 24 h to remove the residual DMSO. The detailed experimental parameters are shown in Table 1.

**Table 1:** Experimental parameters of the dry-jet wet spinning method

Experimental parameters	
Height of air section L (mm)	20
Inner diameter of spinneret d_in_ (mm)	1.4
Outer diameter of spinneret d_out_ (mm)	3.2
thickness of needle tube in spinneret t_in_ (mm)	0.15
Experimental temperature T (°C)	23
Laboratory pressure (kPa)	101.3

### Tensile test

The mechanical properties of the PLGA conduits were characterized using the tensile test using a mechanical tester (AGS-X, Shimadzu, Japan). The SCs and GCs (*n* = 5) were placed in the chuck of the testing machine, and tension was applied at 2 mm/min. The dimensions of the samples were measured using digital calipers (Deli, Ningbo, China) and recorded in the tensile testing software. Young’s modulus was calculated as the slope of the linear region in the 0%–30% strain range on stress–strain curves.

### dECM preparation

An SDS-based optimal decellularization protocol was used based on previously published procedures [27]. For the preparation of the decellularized kidney, Sprague–Dawley (SD) rats were anesthetized with 10% chloral hydrate at a dose of 0.35 mL/100 g. After opening the abdominal cavity, the abdominal aorta and inferior vena cava were fully exposed and ligated. A 24-G indwelling needle was inserted into the abdominal aorta, and 50 mL of 0.01% (w/v) heparin sodium solution was injected at a constant speed to remove the blood until both kidneys turned white. Then, the indwelling needle was connected to a constant flow pump for renal perfusion, where the perfusion contents consisted of 0.01% heparin sodium solution, 1% (w/v) TritionX-100, ddH_2_O, 0.8% (w/v) SDS, PBS enriched with antibiotics (i.e., penicillin, streptomycin, and gentamicin), and amphotericin B. For the preparation of decellularized nerves, the tissues were stirred in 2% (w/v) TritionX-100 for 24 h, then in 1% (w/v) SDS for 48 h, and finally in DI water for 72 h. All solutions were replaced every 8 h, and the decellularized tissues were stored at −20 °C. DNA content was extracted using the TIANamp Genomic DNA Kit and detected using spectrophotometry (Nano Drop One, Thermo Scientific, Waltham, MA).

### Composite nerve conduit fabrication

The same length and weight of decellularized tissue (nerve and kidney) were filled into a SC or GC with approximately 1 cm in length. One end of the decellularized tissue was sutured using 9–0 microsutures, and then, the thread was moved in from the other end of the conduit until the tissue was completely inserted into the conduit.

### SEM

The cross-section of dry GC and SC were prepared, placed on the sample holder, and sputter-coated with gold for 1 min using an ion sputter coater (ISC 150, Supro Instrument, NY, USA). The cross-sectional morphology was observed using SEM (Phenom Pro, Phenom, Netherland).

### Cell culture

PC12 cells were cultured in RPMI-1640 supplemented with 5% FBS and 1% penicillin/streptomycin. The cultures were maintained at 37 °C in a humidified incubator with 5% CO_2_, and the cells in the log phase of growth and within passages P1–P10 were used for experiments. GC and SC were cut from the middle, attached to a 6-well size coverslip, sterilized using 75 v/v% ethanol, and washed with DPBS three times for cell culture. All samples, including the coverslip, decellularized tissues, GC, and SC, were seeded with 200,000 cells per well and cultured for 7 days.

### Live/dead assay

A fluorescence Live/Dead assay was performed based on the manufacturer’s protocol to assess the viability of PC12 on different groups. First, the medium was removed from the 6-well plate and rinsed twice with 1× Assay Buffer. Subsequently, CA and PI were added to the 1× Assay Buffer at a ratio of 1:1000 and 1:400 to prepare reagents for Live/Dead staining. After incubation for 15 min, the Live/Dead assay solution was aspirated, washed with PBS twice, and imaged using a fluorescence microscope (Leica Microsystems, Germany) immediately.

### CCK-8

CCK-8 assays were performed for 7 days to investigate the proliferation of the PC12 on each group. The assays were performed according to the manufacturer’s protocol, where 10 v/v% of CCK-8 working solution was prepared with fully supplemented media and incubated with samples for 2 h. Then, the CCK-8 solution was transferred to a 96-well plate to be observed using a microplate reader (SpectraMax iD3, Molecular Device, Sunnyvale, CA) at 450 nm.

### Phalloidin and DAPI staining

All samples were fixed at 7 days and stained with phalloidin to determine the PC12 cellular morphology on each group. The fixed samples were permeabilized with 0.5% Triton X-100 for 20 min at room temperature. Next, phalloidin solution was applied for further incubation in the dark for 2 h, followed by DAPI staining for the nuclei. Finally, the stained samples were washed with PBS three times and imaged using a fluorescence microscope (Leica Microsystems, Germany).

### Animal experiment

Male adult SD rats, weighing 350 ± 50 g, were purchased from Charles River (Shanghai, China). The animals were kept in the animal care facility of Ningbo University, under a 12-h light and dark cycle, and allowed free access to food and water (serial number: SYXK(Zhejiang)2019-0001). All procedures were performed according to the guidelines of the Animal Care and Use Committee of Ningbo University.

The animals were randomly divided into six groups (*n* = 10 in each group): the autograft group (AG), the GC filled with decellularized nerves group (DN + GC), the decellularized nerves group (DN), the GC filled with decellularized kidney group (DK + GC), the decellularized kidney group (DK), and the GC alone group (GC). All rats were anesthetized with 3%–4% isoflurane (oxygen 0.8–1.5 L/min) inhalation anesthesia. With the rat in the prone position, the right sciatic nerve was exposed with a muscle incision of 2 cm along the ischiofemoral ligament, and a 10-mm tissue was removed after local infiltration anesthesia with 0.1 mL of 2% lidocaine. For the AG group, the removed nerve was rotated 180° and sutured back at each end using 9–0 microsutures. For the GC group, the conduit was secured at both nerve stumps with two stitches using 9–0 microsutures. For the decellularized material groups, the decellularized material was interposed with a nerve gap using the same method. In all groups, the wound was closed in layers using 5–0 nylon sutures (Fig. S1), and the all analysis for the animal experiments were performed 16 weeks after the surgery.

### Sciatic nerve function index (SFI)

SFI is widely used to measure the recovery of motor function after sciatic nerve injury in rats [28]. Sixteen weeks after the operation, the hind limbs of the rats were blackened with ink, and the rats were allowed to walk through a wooden trough with a length of 75 cm, a width of 8 cm, and a height of 15 cm, leaving their hind limb footprints on paper. The foot length (PL), toe spread, and intermediary toe spread of the surgical and contralateral sides were measured. All three measurements were taken from the experimental (E) and normal (N) limbs.

$${{{\mathrm{SFI}}}} = \frac{{109.5\left( {{{{\mathrm{ETS}}}} - {{{\mathrm{NTS}}}}} \right)}}{{{{{\mathrm{NTS}}}}}} - \frac{{38.3\left( {{{{\mathrm{EPL}}}} - {{{\mathrm{NPL}}}}} \right)}}{{{{{\mathrm{NPL}}}}}} + \frac{{13.3\left( {{{{\mathrm{EIT}}}} - {{{\mathrm{NIT}}}}} \right)}}{{{{{\mathrm{NIT}}}}}} - 8.8$$

### Plantar test of thermal stimuli

The SD rats were subjected to the plantar test of thermal stimuli 16 weeks after the operation. The rats were placed on a steel wire mesh platform in the transparent cavity and left to acclimatize for 5 min before testing. A heat source at 60 °C was located 0.5 cm under the hind paws of healthy or surgical rats for a maximum of 20 s. Paw withdrawal latencies were recorded, and three measurements were averaged for each animal. A cutoff latency of 20 s was imposed to avoid tissue damage [29].

### Electrophysiological study

Electrophysiological assessments were performed 16 weeks after the operation. In each group, three rats were randomly selected for the electrophysiological experiments. Each rat was anesthetized, and the sciatic nerve was carefully re-exposed. The ends of a bipolar hook-like stimulating electrode were placed under the sciatic nerve at the proximal ends of the nerve conduit. A needle-like recording electrode was placed in the gastrocnemius muscle. The sciatic nerve was stimulated using a Master-8 Stimulator (A.M.P.I., Israel) at 0.1 mA for 0.2 ms. The compound muscle action potentials (CMAPs) were recorded using the Power Lab System (AD Instruments Pty. Ltd.). Then, the motor nerve reaction latency and the peak amplitude of the CMAPs were calculated [30]. The same procedure was performed on the healthy side, and the percentage of the value of the affected side to the healthy side was calculated.

### Wet muscle weight and Masson’s staining

All gastrocnemii samples (three rats in each group) were harvested under deep anesthesia for evaluation. Both the operated and healthy sides of the SD rats’ gastrocnemius muscles were removed and weighed to assess the extent of atrophy caused by denervation [31]. The harvested gastrocnemius were further fixed with 4% paraformaldehyde, mounted in paraffin, cut to 5-µm-thick sections using a microtome (HistoCore, Leica, Germany), and stained with Masson’s staining based on the manufacturer’s protocol. Briefly, the sections were stained with Weigert-iron hematoxylin for 5 min, differentiated in acidic ethanol differentiation solution for 10 s, immersed in bluing solution for 3 min, and rinsed with DI water. Then, the sections were stained with Ponceau-acid fuchsin solution for 5 min, rinsed with DI water, differentiated with phosphomolybdic acid solution for 1 min, and stained in aniline blue staining solution for 1 min. Finally, the sections were immersed in acetic acid working solution for 1 min, dehydrated with ethanol, transparented by xylene, and sealed with resinene. The samples were imaged at 10× magnification using an inverted microscope (Leica Microsystems, Germany), and the muscle fiber areas were measured with ImageJ. The area was assessed by measuring three images per sample.

### Transmission electron microscope (TEM)

TEM examinations were performed to observe the detailed structure of the axons and myelin sheaths of the regenerated nerves inside the nerve conduits [32]. Three ultra-thin sections from each specimen were obtained using the Leica EM UC7 Ultramicrotome, and the sections were stained with uranyl acetate and alkaline lead citrate for 5 and 10 min, respectively. Then, the stained sections were observed using a TEM (H-7650, Hitachi, Japan). Three middle-powered fields (×5000) in each section were randomly chosen for the quantitative analysis. The thickness of the myelin sheaths was analyzed using ImageJ.

### H&E staining

The nerve tissues were harvested, fixed with 4% paraformaldehyde, mounted in paraffin, cut to 5-µm-thick sections using a microtome (HistoCore, Leica, Germany), and stained with H&E based on the manufacturer’s protocol. Briefly, the sections were baked in an oven at 60 °C for 30 min, deparaffinized with xylene, and washed with ethanol. The sections were stained with hematoxylin solution for 10 min, rinsed with DI water, differentiated with differentiation solution for 3 min, and rinsed with DI water twice. Then, the sections were re-dyeing with Eosin Y solution for 1 min, dehydrated with ethanol, transparented by xylene, and sealed with resinene. The samples were imaged at 10× magnification using an inverted microscope (Leica Microsystems, Germany).

### Immunohistochemistry and immunofluorescence staining

The nerve tissues were harvested, fixed with 4% paraformaldehyde, mounted in paraffin, and cut to 10-µm-thick sections using a microtome (HistoCore, Leica, Germany). Sections were placed onto slides, baked in an oven at 60 °C for 30 min, deparaffinized with xylene, and dehydrated by ethanol. The samples were rehydrated with TE buffer in a pressure cooker for 14 min for antigen retrieval. Samples were incubated in blocking buffer (10% BSA in PBS) for 30 min at room temperature then washed with PBS once. Primary antibodies, including anti-S100β (1:200), NF-H (1:200), and NF200 (1:200) were added and incubated overnight at 4 °C. For immunofluorescence staining, samples were washed with PBS three times and secondary antibody, Alexa Fluor 647/488-conjugated secondary antibody (1:200), was added and incubated for 1 h at room temperature. Samples were washed with PBS three times, mounted with a coverslip using antifade medium containing DAPI, and imaged using a fluorescence microscope (Nexcope, Ningbo, China). For immunohistochemistry staining, samples were washed with PBS three times, incubated with goat anti-mouse antibody (1:200) for 1 h at room temperature, washed three times with PBS, and incubated with HRP conjugates for 30 min. DAB substrate solution was further used for color development, and samples were dehydrated with ethanol, cleared with xylene, mounted with coverslip, and imaged using a light microscope (DMi1, Leica, Germany).

### Statistical analysis

Data were expressed as means ± standard deviations. Statistical Package for the Social Sciences, version 21.0, was used for all statistical analyses. Data of at least three specimens from each experiment were included in the statistical analysis. Statistical significance was identified using the one-way analysis of variance test. The probability level was considered significant at *p* < 0.05.

## Results

### Material characterization of composite nerve conduits

**Fig. 2:**
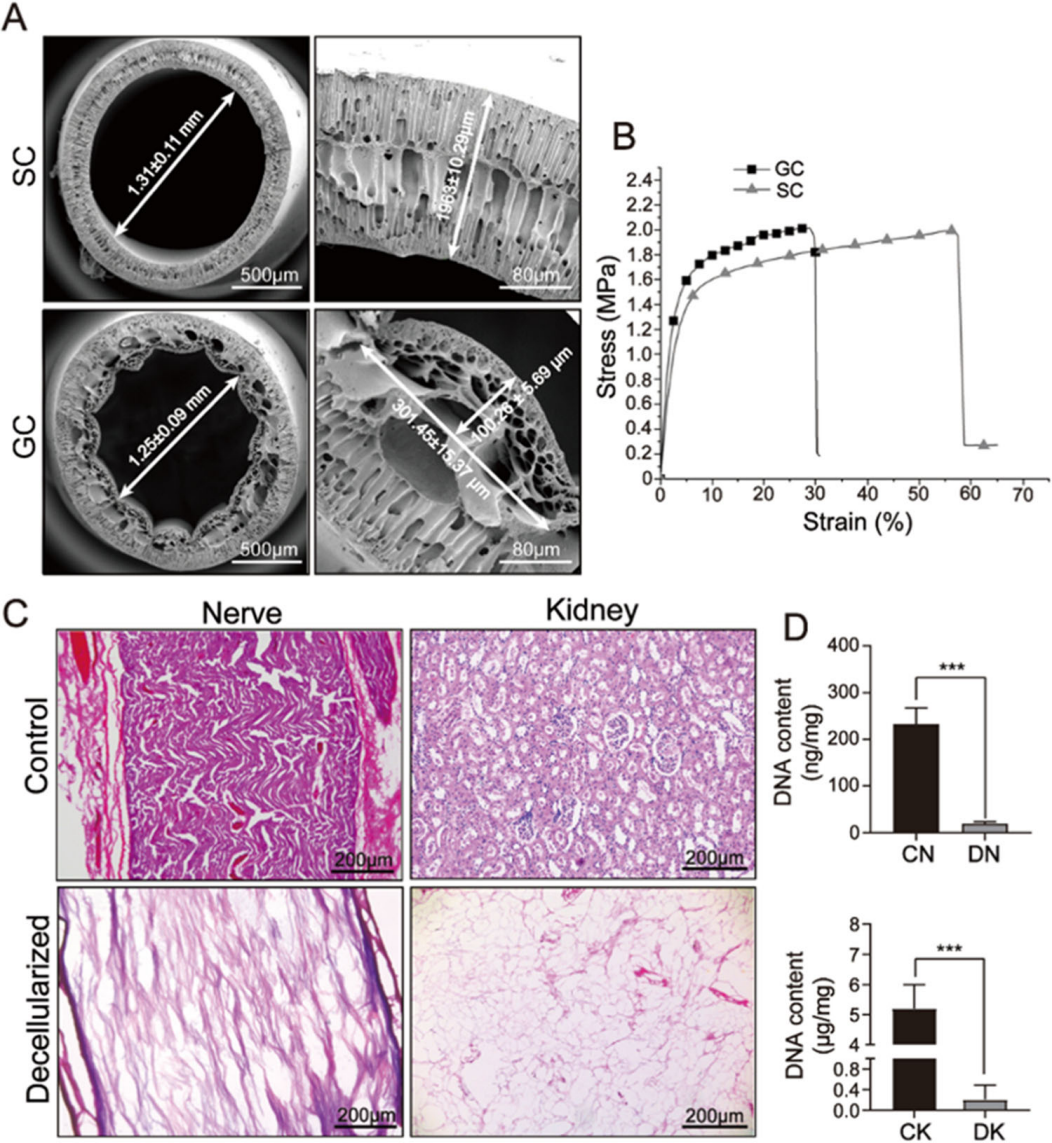
Material characterization of composite nerve conduit. **A** SEM images of smooth and grooved nerve conduit. **B** Tensile tests for smooth and grooved nerve conduit. **C** H&E staining of before and after decellularization. **D** DNA content measurements before and after decellularization. ***p < 0.001

The cross-section of the PLGA hollow tube was imaged using SEM (Fig. 2A), where the SC and GC had an inner diameter of 1.31 ± 0.11 mm and 1.25 ± 0.09 mm, respectively. The outer diameters were 1.52 ± 0.15 mm and 1.45 ± 0.13 mm for SC and GC, respectively. The longitudinal micro-grooved features were also determined, where the groove height and width were 100.26 ± 5.69 μm and 352.26 ± 20.69 μm, respectively. Young’s modulus for SC and GC was evaluated using the tensile test (Fig. 2B). GC was stiffer than SC, with a stiffness of 67.86 ± 6.49 MPa and 52.17 ± 4.94 MPa, respectively, demonstrating the higher deformation resistance for GCs in vivo. H&E staining and DNA content determination were performed to evaluate the cellular removal rate of the rat nerve and kidney (Fig. 2C, D). Compared with the control group, there were almost no cells observed on the decellularized nerve and kidney, and the DNA contents were also significantly reduced, suggesting that cells were successfully removed and only the extracellular matrix was left.

### Cytotoxicity analysis for PLGA conduits and decellularized tissues

**Fig. 3:**
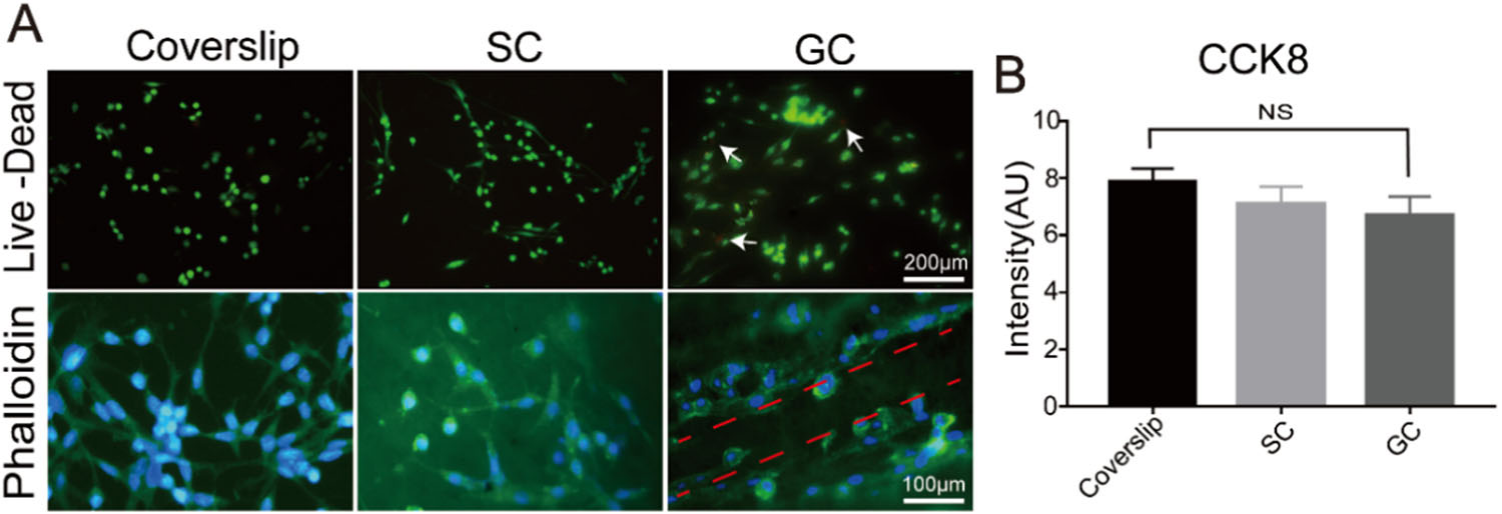
Cytotoxicity analysis for PLGA conduits. **A** Live/Dead assay and phalloidin staining for PC-12 cells cultured on coverslips, SC and GC at day 5. **B** Cell proliferation analysis for PC-12 cells on coverslips, SC and GC at day 7

During the fabrication process of the PLGA conduits and dECMs, toxic chemical components were used. Therefore, the cytotoxicity of the composite nerve conduits should be analyzed in vitro before animal experiments. PC12 cells were seeded on PLGA conduits and decellularized tissue slices separately for 7 days. The cell viability for PC-12 cells on SCs and GCs was analyzed using Live/Dead assays on the day 5, and a coverslip was used as the control group. As shown in Fig. 3A, PLGA grooved conduits had low toxicity, where almost no dead cells were observed in all samples, suggesting that soaking in DI water for 24 h could successfully remove the DMSO that used as solvents in the fabrication process for PLGA grooved conduit. Furthermore, the cellular morphology was determined using phalloidin on day 5, where PC-12 cells demonstrated an elongated shape on all samples, and note that the skeleton direction of PC-12 cells on GC was aligned as the direction of the groove structure, which could promote cellular migration during in vivo nerve regeneration. The CCK-8 assay was further used to determine the viability and growth of PC-12 cells, where the coverslip had the highest cell number on day 7, followed by SCs and GCs, while no significant differences were observed between the two experimental samples (Fig. 3B). These results suggest that both PLGA SCs and GCs could successfully support cellular growth, which could be used for further animal experiments.

### Results of clinical observation

Based on the in vitro cell culture, GCs could physically guide the nerve cell axon direction, while SCs induced a random orientation of nerve axon regeneration and could further result in an inaccurate target reinnervation. Therefore, GCs were selected to prepare the composite nerve conduits containing DN or DK and compared with DN and DK alone for rat sciatic nerve regeneration. Autografts and GCs were used as the control groups.

#### Motor function analysis

**Fig. 4:**
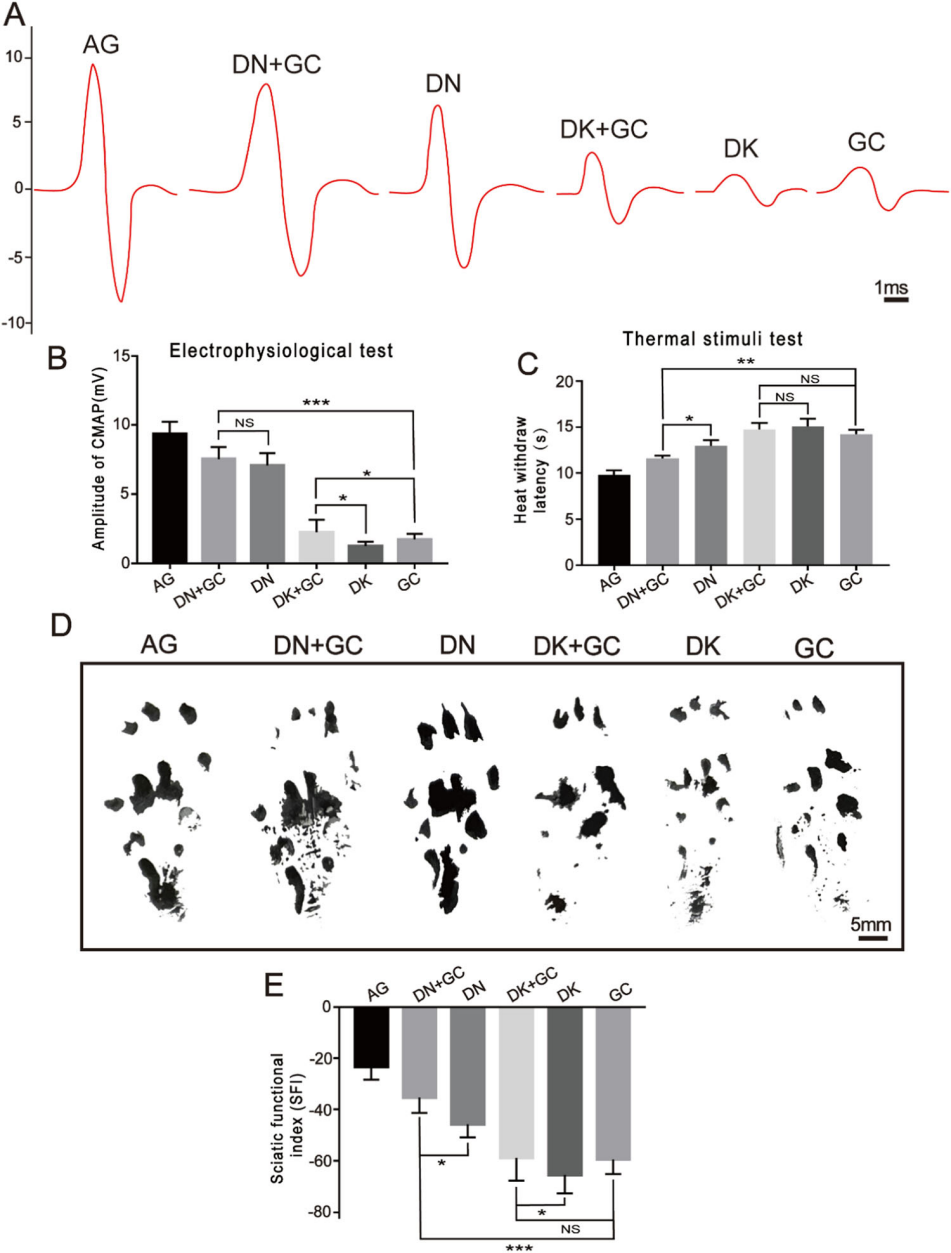
Motor function analysis. **A** Electrophysiological detection for the target muscle. **B** Amplitude of gastrocnemius CMAPs. **C** Thermal reaction latency measurements. **D** The footprints of rat at 16 weeks after the operation. **E** SFI quantification for the footprint at 16 weeks after the operation. AG was significantly different compared with all other groups. *p < 0.05, **p < 0.01, ***p < 0.001

The functional recovery and reinnervation of the regenerated nerves were assessed using electrophysiological detection 16 weeks after the operation (Fig. 4A). The amplitude of the CMAPs was analyzed and compared between all groups (Fig. 4B), where the AG, DN + GC, DN, DK + GC, DK, and GC groups had amplitudes of 9.36 ± 0.87 mV, 7.53 ± 0.86 mV, 7.10 ± 0.88 mV, 2.24 ± 0.91 mV, 1.25 ± 0.31 mV, and 1.72 ± 0.42 mV, respectively. The CMAP amplitude for the AG group was the highest among all groups, whereas the DN + GC group had a CMAP amplitude close to that of the AG group. Among the experimental groups, the CMAP amplitude of samples containing DN was significantly higher than that of samples containing DK. The DN + GC group had a slightly higher CMAP amplitude than the DN group; however, the difference was insignificant. For samples containing DK, the inclusion of GCs significantly increased the CMAP amplitude.

The recovery of sensory function could also be assessed using thermal reaction latency tests. The reaction latency for the AG, DN + GC, DN, DK + GC, DK, and GC groups were 9.83 ± 0.48 s, 11.62 ± 0.28 s, 12.95 ± 0.63 s, 14.74 ± 0.71 s, 15.08 ± 0.83 s, and 14.23 ± 0.48 s, respectively (Fig. 4C). Similar to the trend observed for the CMAP amplitude, the AG group had the lowest latency time among all groups. Samples containing DN had significantly shorter reaction time than samples containing DK. The GCs significantly decreased the latency of samples containing DN, while they did not have effects on samples containing DK.

The rat walking ability was further analyzed and quantified using the SFI. The footprints 16 weeks after the operation showed that the extension of the paw for the surgical side of the AG, DN + GC, DN, DK + GC, DK, and GC groups shortened (Fig. 4D), indicating that the decreased degree of neurological function recovery. The SFI values for the AG, DN + GC, DN, DK + GC, DK, and GC groups were −23.39% ±4.88%, −35.31% ± 5.94%, −45.62% ± 5.18%, −58.90% ±8.81%, −65.5% ± 7.20%, and −59.44% ±5.71%, respectively (Fig. 4E). The AG group still had the best degree of nerve regeneration, and samples containing DN had better recovery of motor function than samples containing DK. Additionally, the GCs further promoted the motor function recovery of DN and DK.

These results demonstrated that DN could promote the regeneration of nerve conduction ability, thermal reaction, and motor function better than DK, and the inclusion of GC could also further promote the recovery effects of DN and DK.

#### Gastrocnemius recovery evaluation

**Fig. 5:**
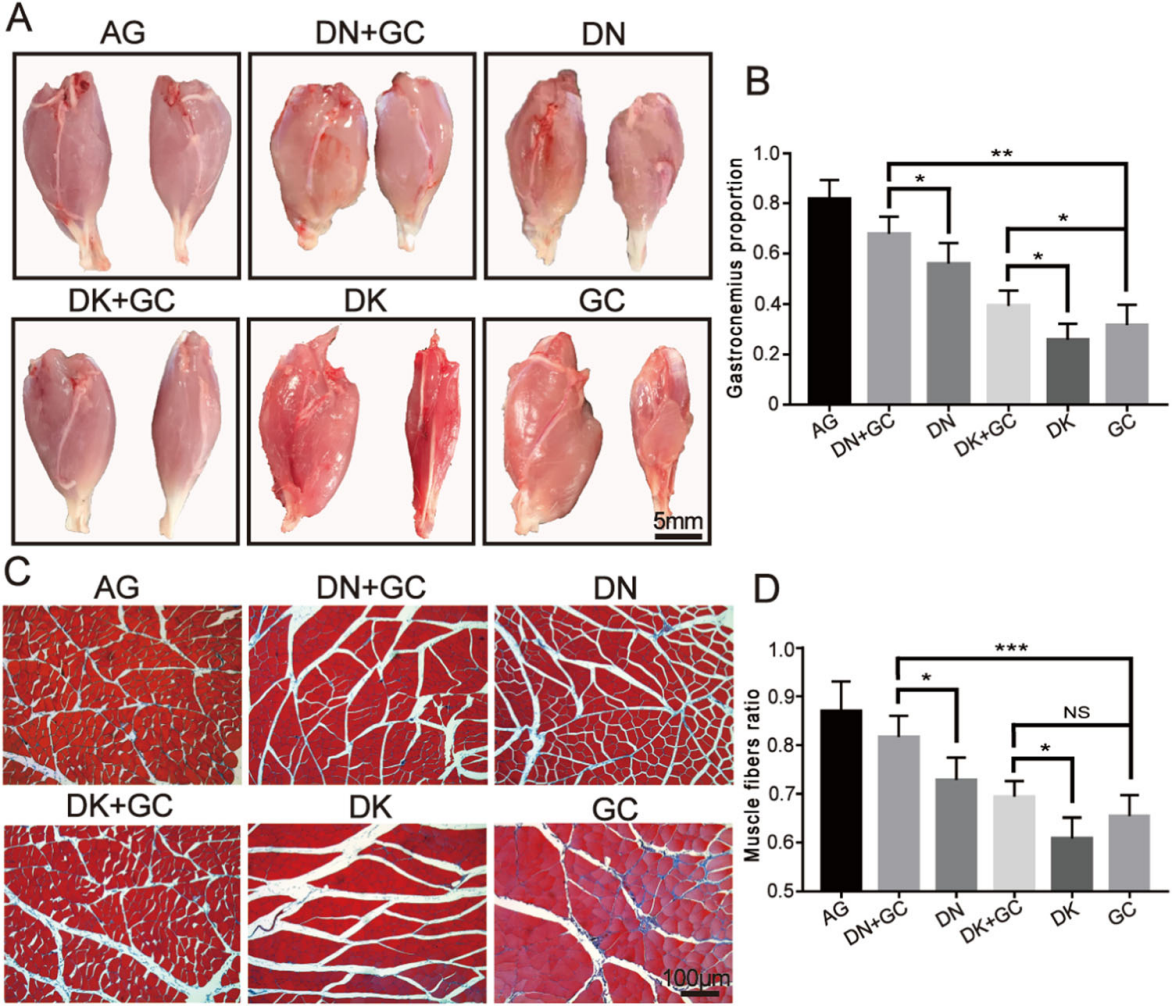
Gastrocnemius recovery evaluation. **A** The pictures of harvested gastrocnemius at 16 weeks after operation. **B** Gastrocnemius proportion calculated using the weight of the operated side divided by that of the normal side. **C** Masson’s staining of gastrocnemius muscle at operation side. **D** Percentage of muscle fibers ratio. AG was significantly different compared with all other groups. *p < 0.05, **p < 0.01, ***p < 0.001

The rats were euthanized after electrophysiological detection, thermal reaction latency tests, and walking ability analysis to harvest the gastrocnemius muscle. As shown in Fig. 5A, the gastrocnemius on the surgical side (right) was still smaller than that on the normal side (left) in each group 16 weeks after the operation, suggesting that there had significant muscle atrophy in the surgical gastrocnemius. The recovery of gastrocnemius was calculated using the weight of the operated side divided by that of the normal side. The AG, DN + GC, DN, DK + GC, DK, and GC groups had the following proportions: 81.60% ±7.80%, 67.8% ±6.87%, 56.0% ±8.19%, 39.4% ±5.98%, 25.8% ±6.38%, and 31.6% ±8.20%, respectively (Fig. 5B). The AG group had the best gastrocnemius muscle recovery rate. Among all experimental groups, the gastrocnemius muscle recovery for samples containing DK was significantly better than that of samples containing DK. The DN + GC group had a significantly better recovery rate than the DN group, which was also observed in the DK group.

The gastrocnemius samples were subsequently stained with Masson’s staining to examine the distribution of muscle and collagen fibers in the gastrocnemius on the surgical side for each group, where the muscle fibers were stained in red and the collagen fibers were stained in blue. The AG, DN + GC, and DN groups had tighter arranged red muscle fibers and less blue collagen fibers than the DK + GC, DK, and GC groups (Fig. 5C). The percentage of muscle fibers was also quantitatively analyzed using ImageJ. The AG, DN + GC, DN, DK + GC, DK, and GC groups had the following proportions: 86.90% ±6.13%, 81.6% ±4.39%, 72.8% ±4.60%, 69.4% ±3.21%, 60.8% ±4.32%, and 65.4% ±4.33%, respectively (Fig. 5D). Similar to the gastrocnemius muscle recovery results, the muscle fiber recovery rate of the AG group was the highest among all groups. Muscle fiber recovery in samples containing DK was significantly higher than that in samples containing DK. The DN + GC group had a significantly better recovery rate than the DN group, which was also observed in the DK group.

Overall, AG as the gold standard method for nerve regeneration significantly promoted the generation of the gastrocnemius muscle. Among the experimental groups, samples containing DN performed better than samples containing DK, suggesting that DN could support the recovery of the gastrocnemius muscle better than DK. The inclusion of GC in samples containing DN and DK further promoted recovery, indicating the promotion effect of the PLGA conduit.

#### Morphological and histological assessments of nerve regeneration

**Fig. 6:**
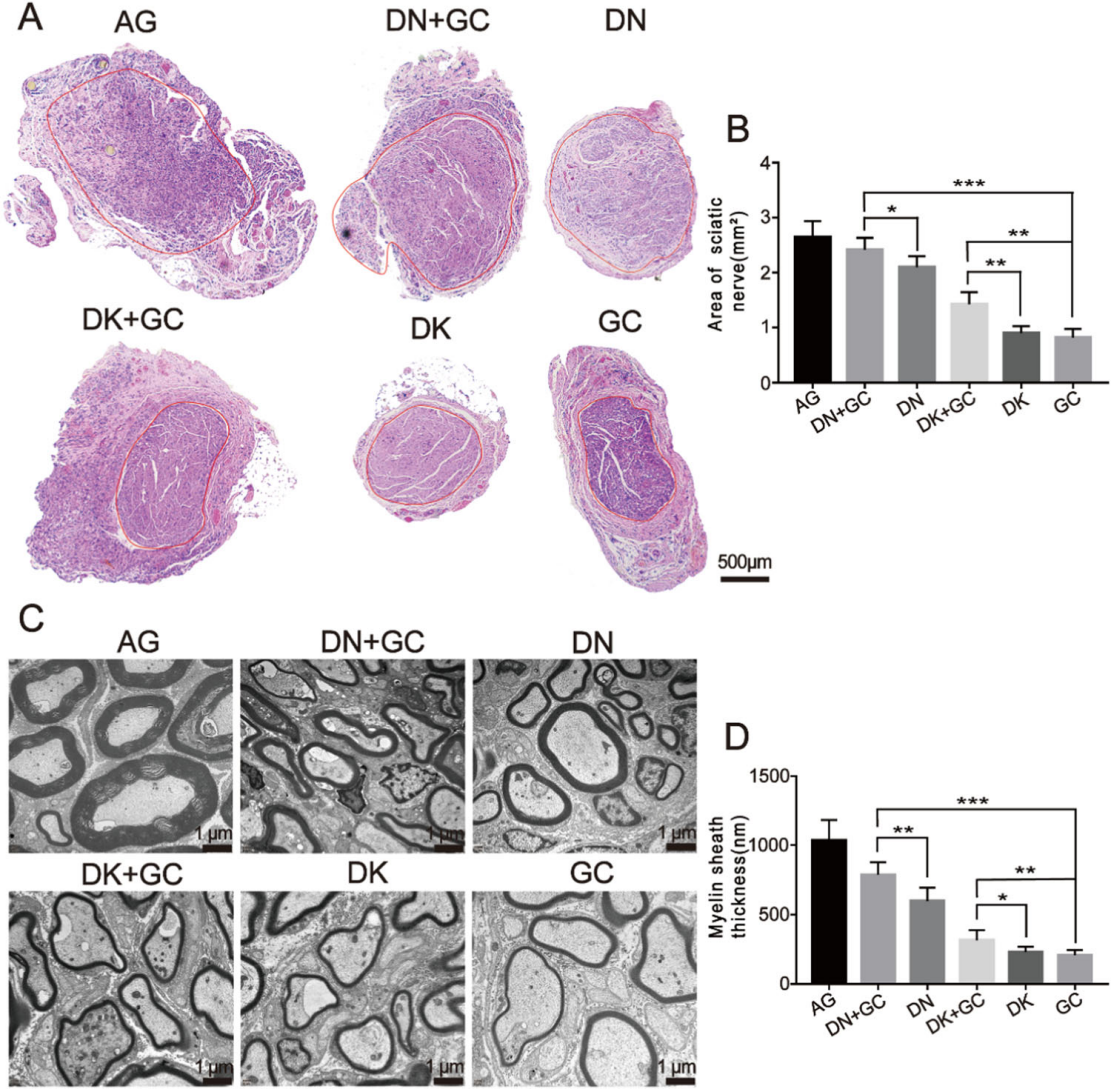
Morphological and histological assessments of nerve regeneration. **A** H&E staining of cross-section areas for regenerated sciatic nerve. **B** Calculation of the area for regenerated sciatic nerve. **C** TEM images for sciatic nerve axons. **D** Myelin sheath thickness measurements. AG was significantly different compared with all other groups. *p < 0.05, **p < 0.01, ***p < 0.001

The regenerated sciatic nerves were also harvested 16 weeks after the operation, and the cross-section areas were stained with H&E to further assess the sciatic nerve recovery, where the nerve fiber areas were selected, as shown in Fig. 6A (red circle). The nerve fiber areas were further measured using ImageJ (Fig. 6B). The AG group had the largest regenerated nerve fiber area, which was close to 3 mm^2^. The DN + GC, DN, DK + GC, and DK groups had a fiber area of 2.42 ± 0.22 mm^2^, 2.10 ± 0.20 mm^2^, 1.43 ± 0.22 mm^2^, and 0.90 ± 0.13 mm^2^, respectively. Obviously, the samples containing DN had significantly larger nerve fiber area than the samples containing DK. The combination with GCs significantly improved never fiber regeneration.

The ultrastructure of the regenerated sciatic nerve axons was also imaged using TEM (Fig. 6C) 16 weeks after the operation, and the thickness was further measured. As shown in Fig. 6D, the AG group still had the thickest myelin. The DN + GC and DN groups had thicker myelin than the DK + GC and DK groups, and the inclusion of GC significantly increased the thickness of myelin. These results indicate that DN had better effects on nerve fiber and myelin regeneration than DK, and the combination of GCs had a positive effect on nerve regeneration.

**Fig. 7:**
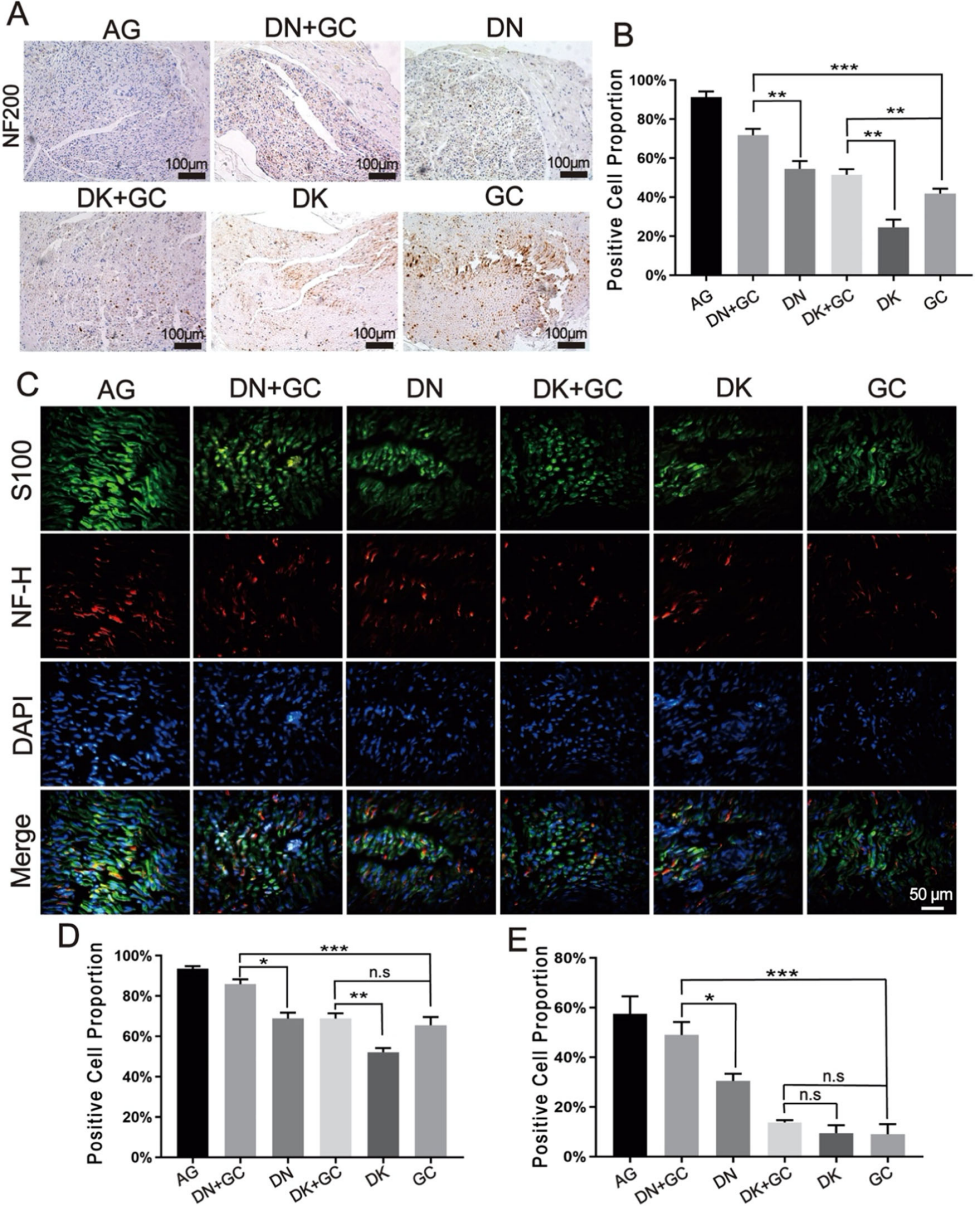
Immunohistochemical and immunofluorescence staining for the nerve regeneration related protein. **A** Immunohistochemical staining for NF200. **B** Proportion of NF200 positive cells by normalizing to total cell number. **C** Immunofluorescence staining for S100 and NF-H. **D**, **E** Proportion of S100β and NF-H positive cells by normalizing to total cell number. AG was significantly different compared with all other groups. *p < 0.05, **p < 0.01, ***p < 0.001

Immunohistochemical and immunofluorescence staining was further used to analyze the protein expression related to nerve regeneration. All groups were stained with NF200, NF-H, and S-100β, and the number of positively stained cells was calculated using ImageJ (Fig. 7). The AG group had the highest number of NF200-positive cells. The samples containing DN had a higher number of NF200-positive cells than the samples containing DK, and the inclusion of GC further increased the number of NF200-positive cells than decellularized tissues alone, indicating an improved axon and Schwann cell growth during nerve regeneration. The AG group had the highest number of S100-β-positive and NF-H-positive cells among all groups. The samples containing DN had a higher number of positive cells than the samples containing DK, and the inclusion of GC could further increase the number of positively stained cells than DN alone, suggesting the improved axonal regeneration and cell differentiation after surgery.

## Discussion

The clinical needs to effectively repair large PNIs using nerve conduits has become a major focus of research in tissue engineering [33]. This has led to the development of various approaches to improve the regenerative capacity of artificial nerve conduits. In our previous study, we developed PLGA GCs and found that they can promote peripheral nerve regeneration better than SCs by introducing physical guidance cues; however, they still have significant weakness compared with autografts because of the lack of biochemical guidance [22]. Therefore, we inserted rat dECMs, including DN and DK, into PLGA conduits to prepare a composite nerve conduit to enhance the nerve regeneration quality.

Both SC and GC developed in this study had an inner and outer diameter close to the diameter of the rat sciatic nerve [34] and the compatibility was further verified with PC-12 cell culture, where the skeleton direction of PC-12 cells on GC was aligned as the direction of the groove structure, which could promote cellular migration during in vivo nerve regeneration and was corresponding to our previous study [21, 22]. Therefore, GCs were selected to prepare the composite nerve conduits containing DN or DK for the following experiments.

The motor function of the regenerated nerves was assessed using electrophysiological, thermal reaction latency tests, and SFI after 16 weeks of surgery. The amplitude of the evoked CMAPs in electrophysiological measurement was directly proportional to the number of regenerated motor nerve fibers [35]. Thermal reaction latency tests were commonly used to evaluate the recovery of sensory function [36]. SFI was used to analyze the rat walking ability of rats after surgery [37]. 0 indicates good function and −100 indicates the complete loss of motor function. Among all groups, AG demonstrated the best recovery, followed by DN + GC, DN, DK + GC, DK, and GC, suggesting that DN could better support nerve regeneration compared with DK, and the combination of PLGA conduit with dECM had a coupling effect on motor function regeneration.

The rats were euthanized after motor function analysis and the gastrocnemius muscle and sciatic nerves were harvested for the following evaluations. Sciatic nerve injury usually triggers a corresponding atrophy of the gastrocnemius muscle, which results in a loss of muscle weight. In this study, the weight of the muscle was measured and the collagen fibers distribution was also stained with Masson’s staining. Similar results as motor function were also found, where the combination of GC and dECM had a better promotion effect on nerve regeneration. The harvested sciatic nerves were used for HE staining, myelination thickness measurement and immunostaining. Myelination plays a critical role in the rapid transmission of nerve signals and is an important tool for assessing nerve recovery [1]. The axon is a critical component of nerves, and its elongation and myelination reflect the level of recovery after surgery [38]. The protein expression that related to nerve regeneration, including NF200, NF-H, and S100 for sciatic nerves was analyzed with immunohistochemical and immunofluorescence staining. NF200 is a marker of myelinated A fiber neurons in rodent dorsal root ganglions (DRGs) [39, 40], which represents regenerated neurofilaments and axons [41] and is expressed in all DRG neurons in humans [42]. S100-β is a calcium-binding protein found in glial cells [43], which has various homeostatic activities, including the regulation of cell proliferation and differentiation [44]. The immunofluorescence staining of S-100β could be used to assess the proliferation of Schwann cells, which is important for successful axonal regeneration after surgery [45]. NF-H is the major component of the neuronal cytoskeleton and primarily functions to provide structural support for axons and regulate axonal diameter [46]. The AG group still had the best nerve recovery results, where it had largest regenerated nerve fiber area, thickest myelination, highest number of NF200- S100-β- and NF-H-positive cells among all groups. In addition, the samples containing DN better supported nerve regeneration compared with DK, and the inclusion of GC had a coupling effect on nerve regeneration.

Based on the results discussed above, GC + DN and DN performed better than GC + DK and DK, respectively. DK was chosen in this study to compare with DN due the enrichment of erythropoietin (EPO) that could promote the regeneration in optic nerve [24] and peripheral nerve [25], which could explain why DK + GC performed better than GC alone in electrophysiological detection, gastrocnemius proportion measurement, and histological assessments. However, based on our previous study [47], DK had much lower expression of brain-derived neurotrophic factor (BDNF), glial cell line-derived neurotrophic factor (GDNF), and fibroblast growth factor 1 (FGF1) compared with DN. All these growth factors have critical influence on the promotion of nerve cell migration, proliferation, survival, outgrowth of sensory, sympathetic, and motor nerve regeneration [48–50], which could have a better promotion effect on nerve regeneration compared with EPO and lead to the better performance of GC + DN compared with GC + DK.

Autograft is the gold standard for peripheral nerve injury providing the most complete regenerative environment for nerve cells [51, 52]. However, autografts still suffer limitations including nerve unavailability, size mismatch, and local tissue adhesion, which caused the urgence to develop artificial nerve conduits that could be used clinically [53–55]. Although the composite nerve conduits developed in this study was still not better than autograft, some of the results were close, like the thermal stimuli test, nerve cross-section area measurement, and S-100β staining for grooved PLGA conduits inserted with decellularized nerve. In addition, this study demonstrated the potential application of decellularized tissues in nerve conduit development, which significantly expand the sources for potential nerve conduit materials, since the decellularized tissues could theoretically be derived from other animals except rat due to the limited allogeneic rejection responses [56]. The idea for the combination of grooved PLGA conduits and decellularized tissues also helped to overcome the weakness of decellularized tissues, like weak mechanical properties and fast biodegradation rate, which pointed a novel direction for nerve conduit development.

## Conclusion

In this study, we designed a novel composite neural conduit. The outer layer was a hollow PLGA GC, and the inner layer was inserted into the acellular rat sciatic nerve and kidney. The material characterization showed that PLGA conduits had enough strength to hold sutures and protect the inserted dECM from external forces. The in vitro cell culture showed that GCs could direct the PC12 axon direction, whereas cells on SCs showed random growth. Therefore, GCs were used to contain DN and DK for developing composite nerve conduits and implanted into the gap of sciatic nerve defects in SD rats. The degree of nerve regeneration was evaluated using the aspects of motor function, gastrocnemius recovery evaluation, morphology, and molecular biology. The combination of GCs with decellularized tissues significantly promoted nerve regeneration compared with the decellularized tissues alone. The DN + GC group had the best promotion effect among all experimental groups, which was close to that of the AG group, suggesting that it is a potential artificial nerve conduit used clinically in the future.

## Supplementary Information

Supplementary Data_revised
