# Supplementary material for: A grooved conduit combined with decellularized tissues for peripheral nerve regeneration
Source: J Mater Sci Mater Med. 2023 Jul 21;34(7):35. doi: 10.1007/s10856-023-06737-z (PMC10361901; doi:10.1007/s10856-023-06737-z)
Supplement: Supplementary file 1 — Supplementary Data_revised [file 10856_2023_6737_MOESM1_ESM.docx]

**A Grooved Conduit Combined with** **Decellularized Tissues for Peripheral Nerve Regeneration**

Enxing Yu^1, *^, Zhiwu Chen^1, *^, Yuye Huang^2^, Yibing Wu^1^, Zonghuan Wang^3^, Fangfang Wang^3^, Miaoben Wu^4^, Kailei Xu^1, 2, 5, 6, 7 #^, Wei Peng^1, #^

^1^Department of Plastic and reconstructive surgery, Ningbo First Hospital, Ningbo 315010, China

^2^Center for Medical and Engineering Innovation, Central Laboratory, Ningbo First Hospital, Ningbo, Zhejiang 315010, China

^3^Central Laboratory, Ningbo First Hospital, Ningbo, Zhejiang, 315010, China

^4^School of Medicine, Ningbo University, Ningbo 315211, China

^5^The State Key Laboratory of Fluid Power and Mechatronic Systems, School of Mechanical Engineering, Zhejiang University, Hangzhou 310028, China

^6^Key Laboratory of 3D Printing Process and Equipment of Zhejiang Province, School of Mechanical Engineering, Zhejiang University, Hangzhou 310028, China

^7^Key Laboratory of Precision Medicine for Atherosclerotic Diseases of Zhejiang Province, Ningbo 315010, China

*These authors contributed equally to this work.

^#^Corresponding authors: weipeng@nbu.edu.cn, xukailei@zju.edu.cn.


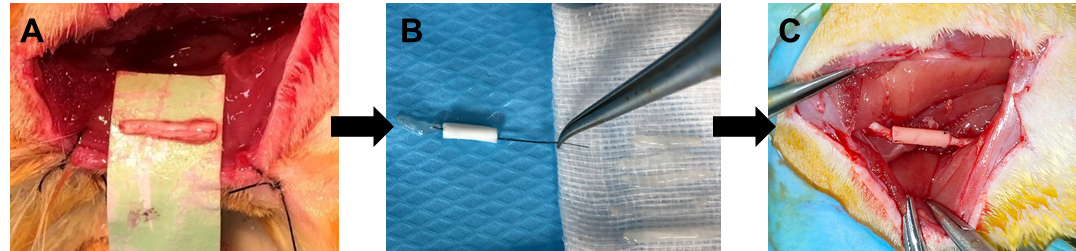


Figure S1. Operation procedure for animal experiments. (A) Sciatic nerve with length of 10 mm was removed. (B) Preparation of grooved conduits inserted with decellularized kidney or nerve. (C) Implantation of composite nerve conduits on sciatic nerve injure.
